# Psychometric Examination of the Abbreviated Version of the Dual School Climate and School Identification Measure-Student (SCASIM-St15) in a Sample of Chilean Adolescents

**DOI:** 10.3389/fpsyg.2021.576051

**Published:** 2021-02-03

**Authors:** José Luis Gálvez-Nieto, Karina Polanco-Levican, Juan Carlos Beltrán-Véliz

**Affiliations:** ^1^Departamento de Trabajo Social, University of La Frontera, Temuco, Chile; ^2^Departamento de Psicología, Temuco Catholic University, Temuco, Chile; ^3^Departamento de Educación, University of La Frontera, Temuco, Chile

**Keywords:** school climate, adolescence, school identification, school, validity, reliability

## Abstract

School climate is a multidimensional construct that has been related to a series of psychological, social, and school variables. The dual school climate and school identification measure-student (SCASIM-St) is a measure that has a multidimensional factor structure, with four first-order factors and a second-order factor, plus an independent factor that evaluates school identification. However, the SCASIM-St is long, with 38 items measuring school climate. The first objective of this study was to examine the psychometric properties of reliability and validity of the abbreviated version of the dual school climate and school identification measure-student (SCASIM-St-15), for use in contexts with time limitations or for explanatory studies that need to apply multiple instruments simultaneously. The second objective was to analyze the degree of invariance for the groups: sex, type of education, and age. The sample was made up of 2,044 students of both sexes (49.1% women and 50.9% men), with a mean age of 14.64 (SD = 0.718), representing 27 secondary schools in Chile. The results indicated that the SCASIM-St15 presents adequate indicators of reliability and construct validity. Evidence of external criterion validity confirmed significant associations with the Attitudes to Institutional Authority in Adolescence Scale measure. The results of the factorial invariance analysis indicate that the SCASIM-St15 remains stable up to the level of metric invariance for the variable sex and the level of scalar invariance for the variables type of education and age. The study concluded that despite the significant decrease in the number of items, the SCASIM-St15 measures school climate in a reliable and valid way, without losing its theoretical and conceptual robustness.

## Introduction

School climate is a very relevant construct in the academic environment and in society in general. Various studies have shown that positive school climate significantly contributes to psychosocial adjustment and decreased risk behaviors (Thapa et al., 2013). A global definition of school climate defines it as the “quality and character of school life. School climate is based on patterns of people’s experiences of school life and reflects norms, goals, values, interpersonal relationships, teaching and learning practices, and organizational structures” (Cohen et al., 2009, p. 182).

School climate is a very relevant construct since a positive climate promotes better academic performance (Li et al., 2020; Trinidad, 2020), prosocial behavior (Villardón-Gallego et al., 2018), self-esteem (Coelho et al., 2020), and identification with the school (Carroll et al., 2017). On the other hand, a toxic school climate is associated with bullying, for example, conflictive teacher-student relationships have a positive effect on bullying, regardless of students’ social level (Longobardi et al., 2018), likewise, low levels of school safety and deteriorated student-student good relationships are positively associated with bullying (Xu et al., 2020). Another study indicates that school climate, through its dimensions, student-student relationships and teacher-student relationships, is indirectly related to bullying, through mediating effects of bystanders’ responses (Cui and To, 2020). In another line of research, deteriorated school climate is a risk factor for school discriminatory bullying in homeless students (Moore et al., 2020). A cross-sectional study applied to 2,560 schools in the United States (Sulak, 2018), related school climate problems to structural factors such as the geographic location and size of the school.

School climate is related to respect for the rules and good relationships with teachers. This study evaluated as a convergent measure the attitude toward authority, which is defined as the degree of importance that adolescents attribute to formally established authority figures, school regulations, and authority figures such as the police (Bonilla et al., 2017). Respect for authority figures inside and outside of educational establishments is related to favorable behavior in other social contexts in which students participate (Garaigordobil, 2016), such as positive relationships between teachers and students (Gálvez-Nieto et al., 2020a) and prevention of school violence (Bonilla et al., 2017). Students who present transgressive attitudes toward authority figures are more likely to engage in cyberbullying (Ortega-Barón et al., 2017) and filioparental violence (Martínez-Ferrer et al., 2018; Del Moral et al., 2019).

## School Climate: A Multidimensional Construct

Before defining school climate, it is important to point out that the international literature presents a wide variety of conceptualizations. For example, Ramelow et al. (2015) conducted a substantial literature review of 4,967 articles between 2003 and 2013, revealing that only four instruments that measured school climate had theoretical foundations. Meanwhile, a literature review conducted by Rudasill et al. (2018) revealed a lack of consistent conceptual and theoretical approaches to the study of school climate. Another literature review (Wang and Degol, 2016), indicated that researchers adopt a large number of conceptualizations that range from theoretical and abstract definitions to very concrete and operational definitions, highlighting that the diversity of definitions makes it difficult to identify the correct measurement factors. After focusing on the reviews that incorporated more evidence and had more methodological robustness (Cohen et al., 2009; Thapa et al., 2013; Wang and Degol, 2016; Rudasill et al., 2018), it was concluded that school climate is a complex construct, which must be measured from a multidimensional perspective.

Given the literature and theoretical framework that supported this study, school climate was defined as the relationship between social and organizational factors. Some of these factors included the relationships between school community members, shared values and standards, personal development through school connection and the emotional growth of the educational community members (Lee et al., 2017). In this definition, school identification is of vital importance because it allows students and the rest of the educational community to develop a sense of belonging and connection with their school (Konold et al., 2017; Lee et al., 2017).

## Measurement of School Climate

Measuring the school climate is a desirable goal both for academic research and for educational establishments, as this idea focuses on the possibility of intervening and improving the school environment. According to a recent systematic review (Lewno-Dumdie et al., 2019; Grazia and Molinari, 2020; Marraccini et al., 2020), the literature on school climate measurement includes a variety of definitions, dimensions, and measures that do not permit a general consensus. For example, the study by Marraccini et al. (2020) conducted a full-text review of 446 articles that identified 26 instruments for measuring school climate and concluded that the identified measures of school climate came from a variety of theoretical backgrounds that captured various constructs and were adapted to different educational levels. According to the review study carried out by Grazia and Molinari (2020), most of the validated school climate scales were only used in one study, showing a fragmented field of study that offers low comparability of results. They also note that among the most widely used instruments are My Class Inventory (Cance et al., 2015; Batanova and Loukas, 2016; Mucherah et al., 2018) and the School Climate-Revised (Suldo et al., 2013; Hendron and Kearney, 2016; Holfeld and Leadbeater, 2017).

The scales with the greatest psychometric evidence to measure the school climate in Chile are the Questionnaire to Evaluate the Social Climate of the School Center (Gálvez-Nieto et al., 2015, 2017) and the school climate scale (López et al., 2014). Although these instruments are linked to psychometric studies that support their relevance and use, their theoretical structures are not very adequate in terms of coverage and content of dimensions. According to what has been discussed in most robust review studies, school climate constructs must present multidimensional structures (Thapa et al., 2013; Wang and Degol, 2016; Rudasill et al., 2018; Lewno-Dumdie et al., 2019; Grazia and Molinari, 2020).

The present study is based on the measure of the dual school climate and school identification measure – student (SCASIM-St). This instrument has a second-order factor called School Climate which groups five first-order factors, four of them which evaluate the school climate: Student-Student Relations, Student-Staff Relations, Academic Emphasis, Shared Values and Approach. It also presents a fifth factor of the first order - independent of the common factor of the second order - called School Identification (Lee et al., 2017). The SCASIM-St has presented a stable factorial structure in the three countries where it has been applied: Australia, where it was originally designed and applied (Lee et al., 2017), and in Turkey and Chile, respectively (Demirtas-Zorbaz and Hoard, 2019; Gálvez-Nieto et al., 2020b).

The dual measure of school climate and school identification is a self-report measure focused on interpersonal relationships within an educational community that also assesses school identification. This instrument could be a useful measure for socio-educational evaluation and intervention in areas such as individual school adaptation, improvement of school performance and school management. The theoretical structure of SCASIM-St is based on the ecological theoretical model of Bronfenbrenner (2002), who states that individual behaviors are explained by the various social subsystems in which an adolescent develops. The SCASIM-St provides a theoretical framework that integrates the measurements of school climate and school connectedness (Lee et al., 2017) from inputs derived from Tajfel and Turner’s (1979) theory of social identity.

Considering the importance of evaluating the school climate, the scarcity of instruments to measure it and the need to develop an abbreviated version of SCASIM-St, the first objective of this article was to examine the psychometric properties of reliability and validity of the abbreviated version of the dual school climate and school identification measure-student (SCASIM-St-15). The second objective is to analyze the degree of invariance for the groups: sex, type of education, and age.

## Materials and Methods

### Participants

The investigated population included students from public institutions, subsidized private schools and non-subsidized private schools. All participants were secondary education students that lived in one of five macro-zones of Chile, which contain a total of 47,714 students (Ministerio de Educación de Chile, 2019).

Participants were selected through stratified probability sampling with a 95% confidence interval, a variance of *p* = *q* = 0.50 and a standard error of 3% (Scheaffer et al., 1987). The region, the type of education and the administrative dependency of the schools were considered as strata. The sample consisted of 2,044 students (49.1% women and 50.9% men), with an average age of 14.64 (SD = 0.718), representing 27 secondary schools in Chile. While the selected schools included students from various socioeconomic backgrounds, the majority represented low and medium socioeconomic levels.

### Instruments

To achieve the objectives of the study, a sociodemographic questionnaire was applied that collected information about the students’ age, sex, school level, and type of school, among other information.

Simultaneously, the adapted version of the SCASIM-St was applied (Gálvez-Nieto et al., 2020b). The SCASIM-St is a self-report scale that measures school climate and school identification (Lee et al., 2017) based on 38 items written in a positive way (Vigil-Colet et al., 2020) that are answered using a five-point ordinal scale (1 = strongly disagree, 5 = strongly agree). The SCASIM-St has the following factor structure: four first-order factors called Student-Student Relations (seven items, e.g., “Students are friendly to each other”), Student-Staff Relations (nine items, e.g., “Staff care about students”), Academic Emphasis (eight items, e.g.,“Teachers challenge students to do better”), and Shared Values and Approach (eight items, e.g.,“The school values and goals are well understood”). These four factors are grouped into a common factor called School Climate. The SCASIM-St also presents a fifth factor, School Identification (six items, e.g., “I feel a strong connection with this school”), which is related to the second order factor.

In addition, the adapted version of the Attitudes to Institutional Authority in Adolescence Scale (AIA-A) was applied (Gálvez-Nieto et al., 2018). The AIA-A is a self-report scale that assesses adolescent attitudes toward authority figures, has nine items and is answered using a five-point ordinal scale (1 = Never, 5 = Always). The factorial structure of the AIA-A is made up of two factors: Positive Attitude to Authority (five items e.g., “The police are there to make a better society for all”), referring to the degree of respect toward teachers and the police; and Positive Attitude to Transgression (four items e.g., “It is normal to break the law if no one is harmed”), referring to positive attitudes toward transgression of school rules. In this study, the AIA-A factors presented adequate reliability indices. The factor Positive Attitude to Authority obtained a Cronbach’s alpha of 0.745 (McDonald’s omega = 0.759) and an average variance extracted equal to 0.443. The factor Positive Attitude to Transgression obtained a Cronbach’s alpha of 0.762 (McDonald’s omega = 0.777) and an average variance extracted equal to 0.556. The confirmatory factor analysis presented adequate goodness-of-fit indices: *WLSMV* χ^2^ (*df* = 26) = 417.164, *p* < 0.001; CFI = 0.958; TLI = 0.941; RMSEA = 0.064. All factor loadings were statistically significant (*p* < 0.001).

### Process

To create the abbreviated version of the SCASIM-St and maintain the reliability and validity properties, a subset of items was selected considering the following criteria; (a) high statistical performance, that is, high factor loadings, high full-scale item correlation and maximal variability in responses and (b) conceptual considerations such as high face validity, that is, high item-dimension conceptual coherence (Stanton et al., 2002).

Before the application of the surveys, the school directors were contacted and asked to sign an agreement to access the sample of students. Subsequently, informed consents were sent to the parents of the students. Once the parental authorizations were obtained, the students responded to an informed consent. After the ethical principles of the project were approved, the surveys were administered during the first hour of class.

### Analysis of Data

The missing values were less than 5% of the sample and were treated using the multiple imputation method available in MPLUS v.8.1 software (Muthén and Muthén, 2017). Descriptive statistics were analyzed for each of the items. In order to properly select the analysis approach, Kolmogorov–Smirnov univariate normality tests (see Table 1, *p* < 0.001) and multivariate tests (Skewness-test = 406.590; Kurtosis-test = 147.716) were performed. In both cases the tests were statistically significant (*p* < 0.001), suggesting the use of robust estimators in the absence of normality in the data (Table 1).

**TABLE 1:** Descriptive statistics, corrected item-total correlation and confirmatory factor analysis.

**Student-student relationships/Relaciones estudiante-estudiante (AVE = 0.54)**	**Mean**	**Standard deviation**	**Skewness**	**Kurtosis**	**K-S test**	**C.I.T.C**	**CFA**
it1 Students care about each other/Los estudiantes se cuidan unos a otros	3.32	0.933	−0.427	0.270	0.221*	0.635	0.728*
it2 Students are friendly to each other/Los estudiantes son amigables entre si	3.47	0.931	−0.506	0.243	0.236*	0.637	0.721*
it3 Students go out of their way to help each other/Los estudiantes buscan la forma de ayudarse unos a otros	3.31	0.898	−0.423	0.254	0.217*	0.649	0.743*
**it4 Students treat each other with respect/Los estudiantes se tratan con respeto entre si**	2.98	0.960	−0.182	−0.233	0.237*	0.664	0.746*
**it5 Students are fair to each other/Los estudiantes son justos entre sí**	3.10	0.897	−0.218	−0.015	0.242*	0.656	0.753*
**it6 Students show understanding to each other/Los estudiantes muestran comprensión entre ellos**	3.25	0.891	−0.345	0.109	0.220*	0.668	0.754*
it7 Students are accepting of each other’s differences/Los estudiantes aceptan diferencias de los demás	3.48	1.054	−0.468	−0.211	0.212*	0.592	0.699*
Student-staff relationships/Relaciones estudiantes-personal (AVE = 0.61)							
it8 Staff care about students/El personal cuida a los estudiantes	4.00	0.828	−0.908	1.305	0.291*	0.659	0.748*
it9 Staff are friendly to students/El personal es amigable con los estudiantes	3.94	0.866	−0.767	0.735	0.269*	0.688	0.771*
**it10 Staff go out of their way to help students/El personal busca formas de ayudar a los estudiantes**	4.01	0.802	−0.714	0.747	0.274*	0.709	0.826*
it11 Staff treat students with respect/El personal trata a los estudiantes con respeto	4.16	0.787	−1.038	1.728	0.266*	0.709	0.796*
it12 Staff listen to what students have to say most of the time/El personal escucha lo que los estudiantes tienen que decir la mayoría del tiempo	3.72	0.900	−0.511	0.142	0.255*	0.708	0.777*
it13 Staff involve students in decisions and planning/El personal involucra a los estudiantes en las decisiones y planificación	3.53	0.917	−0.391	0.212	0.214*	0.538	0.635*
**it14 Staff are fair in their dealing with students/El personal es justo en su trato con los estudiantes**	3.80	0.891	−0.656	0.506	0.263*	0.747	0.833*
**it15 Staff show understanding to students/El personal muestra comprensión a los estudiantes**	3.84	0.841	−0.711	0.879	0.281*	0.753	0.855*
it16 Staff take students’ concerns seriously/El personal toma en serio las preocupaciones de los estudiantes	3.80	0.935	−0.564	0.093	0.239*	0.706	0.793*
Academic emphasis/Énfasis académico (AVE = 0.58)							
it17 Teachers encourage students to try out new ideas (think independently)/Los profesores animan a los estudiantes a probar nuevas ideas Pensar independientemente)	3.89	0.899	−0.807	0.826	0.264*	0.643	0.754*
**it18 Teachers challenge students to do better/Los profesores desafían a los estudiantes a hacerlo mejor**	4.03	0.816	−0.823	1.014	0.279*	0.686	0.786*
it19 Teachers are willing to give students extra help on school work if needed/Los profesores están dispuestos a dar una ayuda extra en el trabajo escolar si es necesario	3.88	0.904	−0.746	0.641	0.262*	0.609	0.693*
it20 Teachers set high standards for learning in their classes/Los profesores establecen altos estándares de aprendizaje en sus clases	3.87	0.800	−0.513	0.482	0.276*	0.650	0.767*
it21 Teachers expect everyone to work hard/Los profesores esperan que todos trabajen mucho	3.99	0.861	−0.755	0.607	0.261*	0.600	0.657*
**it22 Teachers want every student to do their best/Los profesores quieren que cada estudiante haga su mejor esfuerzo**	4.29	0.812	−1.386	2.631	0.276*	0.729	0.834*
**it23 Teachers believe that every student can be a success/Los profesores creen que cada estudiante puede ser un éxito**	4.10	0.923	−1.04	0.997	0.239*	0.674	0.800*
it24 Teachers give useful feedback/Los profesores dan una retroalimentación útil	3.90	0.875	−0.715	0.686	0.261*	0.656	0.777*
Shared values and approach/Valores y enfoques compartidos (AVE = 0.49)							
it25 Students and staff are working toward the same goals/Los estudiantes y el personal luchan por los mismos objetivos	3.43	0.872	−0.431	0.424	0.226*	0.587	0.707*
**it26 There is a sense that we are all on the same team/Hay un sentido de pertenencia y que todos estamos en el mismo equipo**	3.22	0.971	−0.259	−0.145	0.209*	0.650	0.751*
**it27 There is school spirit and pride/Hay espíritu y orgullo escolar**	3.48	0.938	−0.452	0.177	0.229*	0.616	0.716*
**it28 The school values and goals are well understood/Los valores y objetivos de la escuela son bien entendidos**	3.42	0.927	−0.400	0.070	0.223*	0.648	0.719*
it29 New students and staff are made to feel welcome as part of the group/Los nuevos estudiantes y personal son hechos sentir bienvenidos como parte del grupo	3.74	0.905	−0.559	0.370	0.244*	0.555	0.693*
it30 Student and staff who uphold the values of the school are recognized and celebrated/El estudiante y personal que defiende los valores de la escuela son reconocidos y celebrados	3.58	1.000	−0.544	0.078	0.230*	0.501	0.593*
it31 The expectations and rules are clear/Las expectativas y reglas son claras	3.88	0.901	−0.738	0.609	0.258*	0.582	0.701*
it32 The rules related to discipline are clear and well-understood by staff and students/Las reglas relacionadas con la disciplina son claras y bien entendidas por el personal y los estudiantes	3.69	0.971	−0.544	0.086	0.228*	0.606	0.710*
School identification/Identificación escolar (AVE = 0.49)							
it33 Being a part of this school is important to me/Ser parte de esta escuela es importante para mi	3.69	1.066	−0.725	0.132	0.241*	0.771	0.855*
**it34 I am happy to be a part of this school/Soy feliz de ser parte de esta escuela**	3.65	1.052	−0.622	−0.004	0.227*	0.823	0.908*
**it35 I feel a strong connection with this school/Siento una fuerte conexión con esta escuela**	3.20	1.071	−0.218	−0.390	0.202*	0.824	0.904*
**it36 I identify with this school/Me identifico con esta escuela**	3.24	1.084	−0.293	−0.370	0.199*	0.805	0.888*
it37 I feel I belong at this school/Siento que pertenezco a esta escuela	3.47	1.078	−0.530	−0.176	0.221*	0.781	0.851*
it38 I care about this school/Cuido a esta escuela	3.95	0.931	−1.048	1.394	0.274*	0.494	0.650*

*Items marked in bold were selected to form the final version of the abbreviated scale SCASIM-ST15. C.I.T.C, corrected item-total correlation; CFA, confirmatory factor analysis; AVE, average variance extracted. ^∗^*p* < 0.001.*

The CFA models used the polychoric correlations matrix and the weighted least squares means and variance adjusted (WLSMV) estimation method. Several goodness-of-fit indexes were used to evaluate the CFA models: *WLSMV*-χ^2^, comparative fit index (CFI), Tucker-Lewis index (TLI) and root mean square error of approximation (RMSEA). For CFI and TLI, values equal to or greater than 0.90 were considered reasonable (Schumacker and Lomax, 2016). For RMSEA, values less than or equal to 0.080 were considered a reasonable adjustment (Browne and Cudeck, 1993). Subsequently, a factorial invariance analysis was carried out for the variables sex, type of education, and age. This analysis considers the following models (Vandenberg and Lance, 2000): M0 configural (equal number of factors), M1 metric (equal factor loadings), and M2 scalar (equality of intercepts). The corrected item-total correlation method and the McDonald’s ω and Cronbach’s α coefficients were used to estimate reliability (Green and Yang, 2015; Trizano-Hermosilla and Alvarado, 2016).

## Results

### Descriptive Analysis

The descriptive results considering the mean value of each item showed the highest value for item 22 “Teachers want every student to do their best,” with a mean of 4.29 (SD = 0.812). The item with the lowest mean value was item 4 “Students treat each other with respect,” with a mean of 2.98 (SD = 0.960). In addition, in order to identify the items that would make up the abbreviated version of the SCASIM-St, the results of the corrected item-total correlation, confirmatory factor analysis and a high face validity were analyzed. In Table 1, the items with the highest statistical performance and conceptual representativeness of the dimension are marked in bold. For example, for the first factor, Student-Student Relations, the selected items were 4four, five, and six.

### Full SCASIM-St Validity and Reliability

With the aim of evaluating the factorial structure of the SCASIM-St, a confirmatory factorial analysis was performed with the full 38 item scale. For this, a second-order model was estimated that grouped four factors, plus an independent factor. Goodness of fit indices gave satisfactory results: *WLSMV* χ^2^ (660) = 3,528.580, *p* < 0.001; CFI = 0.964; TLI = 0.961; RMSEA = 0.049 (CI90% = 0.048–0.051). The factor loadings presented satisfactory and statistically significant results (Table 1). These results provided evidence that the model fit the data adequately, confirming the original theoretical structure of the scale. Finally, the average variance extracted (AVE) was estimated, obtaining values that ranged from 0.49 to 0.72 (Table 1).

Once the factorial structure of the SCASIM-St was confirmed, the instrument’s reliability levels were estimated. As can be seen in Table 2, the reliability estimators for the full scale (38 items) presented satisfactory results.

**TABLE 2:** Evidence of reliability.

			**95% Confidence interval**	
**Dimension**	**McDonald’s ω**	**Cronbach’s α**	**Lower**	**Upper**
Student-student relations	0.867 (0.782)	0.866 (0.780)	0.855 (0.762)	0.875 (0.798)
Student-staff relations	0.910 (0.819)	0.908 (0.815)	0.901 (0.800)	0.915 (0.830)
Academic emphasis	0.887 (0.804)	0.885 (0.799)	0.876 (0.782)	0.893 (0.815)
Shared values approach	0.856 (0.781)	0.854 (0.781)	0.843 (0.762)	0.865 (0.798)
School identification	0.914 (0.902)	0.912 (0.901)	0.905 (0.893)	0.918 (0.909)

*The values in parentheses () correspond to the reliability estimates for the abbreviated version of SCASIM-St.*

### Evidence of Validity of the SCASIM-St15

**FIGURE 1:**
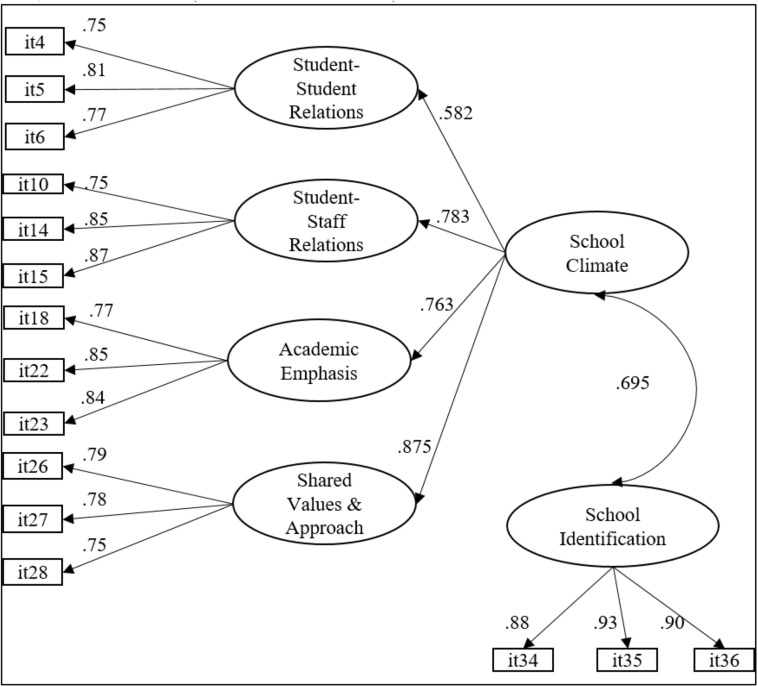
The factorial structure of the abbreviated SCASIM-St. All estimated parameters were statistically significant (*p* < 0.001).

To assess whether the abbreviated structure of the SCASIM-St provided adequate psychometric indicators, a CFA model was estimated with the 15 items on the scale. A second-order model that grouped four first-order factors, plus an independent factor (Figure 1) was evaluated. This model provided an excellent fit: *WLSMV* χ^2^ (*df* = 85) = 611.596, *p* < 0.001; CFI = 0.984; TLI = 0.981; RMSEA = 0.058 (C.I. 90% = 0.054–0.063).

### External Validity Criteria for the Abbreviated SCASIM-St

**FIGURE 2:**
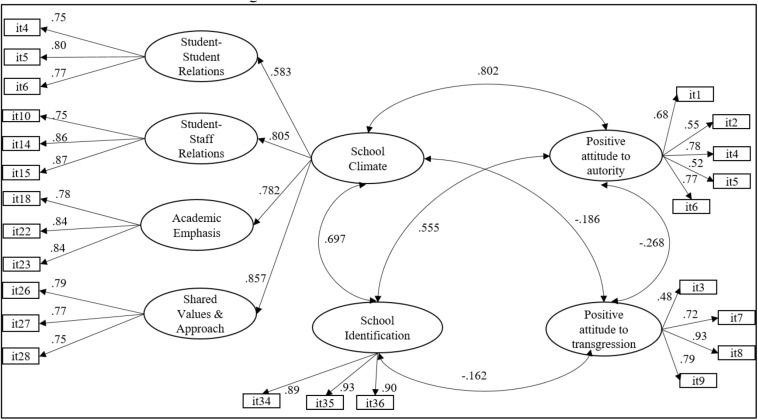
Structural equation model. All the estimated parameters were statistically significant (*p* < 0.001).

To evaluate the relationships between SCASIM-St15 and AIA-A, a structural equation model was evaluated (Figure 2). The results of this model provided satisfactory goodness-of-fit indices *WLSMV* χ^2^ (*df* = 242) = 61,306.242, *p* < 0.001; CFI = 0.973; TLI = 0.969; RMSEA = 0.052 (C.I. 90% = 0.050–0.055) and confirmed that both scales presented significant and positive correlations between School Climate, School Identification, and Positive Attitude to Authority. The School Climate and School Identification factors presented significant and negative correlations with the factor Positive Attitude to Transgression.

### Factorial Invariance

Once the final version of the SCASIMSt15 was obtained a factorial invariance analysis was performed for the variables sex, type of education, and age. The first contrasted model was M0 or configuration invariance, with satisfactory results for the sex variable [χ^2^ (160) = 287.952, *p* < 0.001; CFI = 0.988; TLI = 0.984; RMSEA = 0.029], type of education [χ^2^ (160) = 283.552, *p* < 0.001; CFI = 0.988; TLI = 0.985; RMSEA = 0.029], and age [χ^2^ (160) = 302.765, *p* < 0.001; CFI = 0.988; TLI = 0.984; RMSEA = 0.029]. These results allowed us to conclude that the factorial structure of the SCASIM-St15 is the same, independent of sex, type of education, and age.

Subsequently, the metric invariance model M1 was evaluated, which imposes restrictions on factor loadings. The results indicated that there are no statistically significant differences between the metric and configuration models for sex [χ^2^ (170) = 292.886, *p* < 0.001; CFI = 0.988; TLI = 0.985; RMSEA = 0.028; Δχ^2^ = 4722; Δ*df* = 10; *p* (Δχ^2^) = 0.9089], type of education [χ^2^ (170) = 292.785, *p* < 0.001; CFI = 0.988; TLI = 0.986; RMSEA = 0.028; Δχ^2^ = 8.698; Δ*df* = 10; *p* (Δχ^2^) = 0.5610], and age [χ^2^ (170) = 316.933, *p* < 0.001; CFI = 0.987; TLI = 0.984; RMSEA = 0.029; Δχ^2^ = 13.941; Δ*df* = 10; *p* (Δχ^2^) = 0.1757]. These results allowed us to conclude that the factor loadings of the scale items are equivalent for sex, type of education, and age.

In addition, the degree of scalar invariance (M2) was evaluated, including restrictions in the intercepts of the items. The results indicated that there are no statistically significant differences between the metric and scalar models for the variable type of education [χ^2^ (180) = 309.142, *p* < 0.001; CFI = 0.988; TLI = 0.986; RMSEA = 0.028; Δχ^2^ = 16,189; Δ*df* = 10; *p* (Δχ^2^) = 0.0944] and age [χ^2^ (180) = 325.844, *p* < 0.001; CFI = 0.987; TLI = 0.985; RMSEA = 0.028; Δχ^2^ = 7.012; Δ*df* = 10; *p* (Δχ^2^) = 0.7243]. The sex variable presented statistically significant differences for this model [χ^2^ (180) = 324.037, *p* < 0.001; CFI = 0.986; TLI = 0.984; RMSEA = 0.029; Δχ^2^ = 33.959; Δ*df* = 10; *p* (Δχ^2^) = 0.0002], therefore no differences were estimated for this variable.

Subsequently, differences in latent means were evaluated. The first hypothesis test was carried out using type of education as the grouping variable, and the results showed statistically significant differences for the variable Student-Student Relations [*t*-test (2038) = 3.186; *p* = 0.001; Cohen’s *d* = 0.141] and School Identification [*t*-test (821.544) = 6.462; *p* < 0.001; Cohen’s *d* = 0.451], with the lowest averages obtained by public schools. The second hypothesis test was performed for the variable age, and the results suggested maintaining the null hypothesis, that is, there are no statistically significant differences for the variable age.

### Evidence of Reliability of the SCASIM-St-15

In relation to the evidence of reliability of the abbreviated SCASIM-St15, all indicators presented satisfactory values (Table 2). The factor School Identification had the highest reliability (ω = 0.902), while the factor Shared Values and Approach had the lowest reliability (ω = 0.781).

## Discussion

This study had two objectives. The first objective examined the psychometric properties of reliability and validity of the abbreviated version of the School Climate and School Identification Scale (SCASIM-St15). The second objective was to analyze the degree of invariance for the groups: sex, type of education, and age.

Regarding the first objective, the results indicated that the SCASIM-St15 maintained a second order structure that grouped four factors, plus an independent factor, as well as maintained adequate levels of reliability. These results suggest that indicated that despite having significantly decreased the number of items, the factor structure of SCASIM-St remained stable and was consistent with previous studies (Lee et al., 2017; Demirtas-Zorbaz and Hoard, 2019; Gálvez-Nieto et al., 2020b). To assess the validity of external criteria, the SCASIM-St15 scores were correlated with the AIA-A. The results of this investigation showed that the general factor of the SCASIM-St15 called School Climate and the School Identification factor presented significant and negative correlations with the Positive Attitude to Transgression factor, but significant and positive correlations with the Positive Attitude to Authority factor. These results are consistent with previous studies which suggest that students who present high levels of transgression of norms, in turn present relational problems in schools (Gálvez-Nieto et al., 2018), and violence toward their parents (Martínez-Ferrer et al., 2018; Del Moral et al., 2019). On the other hand, a positive attitude toward the norms of institutions such as the school or the police favors academic success (Trinidad, 2020) and psychosocial adjustment in other social contexts (Bonilla et al., 2017).

Regarding the results of the second objective, this study shows that the factorial structure of the SCASIM-St15 remains stable up to the level of metric invariance for the variable sex and the level of scalar invariance for the variables type of education and age. In addition, differences in latent means were evaluated for type of education and age, and statistically significant differences were found for the variable type of education. Students from public schools obtained lower scores in the Student-Student Relations and School Identification factors. These differences could be explained given that public schools are generally large, an aspect that makes school identification difficult (Carroll et al., 2017), and that they are often located in high crime areas (Sulak, 2018) and unsafe neighborhoods (Gálvez-Nieto et al., 2020a).

The abbreviated version of SCASIM-St15 offers a complete measure of school climate. In terms of coverage, it provides a theoretically robust construct which will facilitate its application in educational contexts. It can also be used by researchers who require the simultaneous application of several instruments in order to lower the response burden on students and considerably decrease resource requirements.

In relation to the implications for educational practice, the SCASIM-St15 is a brief tool for measuring school climate, it provides a valid and reliable instrument that will allow evaluating psychosocial interventions in educational settings. Likewise, the SCASIM-St-15, through its five dimensions, provides a work guide that can help the selection and implementation of pertinent interventions to improve school climate and also reduce the incidence of relevant problems that affect a significant proportion of students, such as bullying. In this line of research, it is necessary to strengthen the capacity of students to understand and deal with bullying, strengthening key dimensions of school climate, such as the relationships between teachers-students and students-students (Longobardi et al., 2018; Xu et al., 2020), from a systemic perspective, include parents in prevention programs, stimulating greater parental supervision, and strengthening positive values to improve the school climate.

The results of this investigation should be interpreted with caution. Although the selected sample represented a wide variety of zones and regions in Chile, it only provided evidence through a cross-sectional design. In this sense, new research should provide more robust psychometric evidence using longitudinal designs. Another limitation of this research is that self-reported instruments were only measured from the perspective of the students. Future studies should consider including new hypotheses about the dimensionality of the scale, considering recent literature and new approaches to data analysis (Garrido et al., 2019).

## Data Availability Statement

The datasets generated for this study are available on request to the corresponding author.

## Ethics Statement

The studies involving human participants were reviewed and approved by Comité de ética de la Universidad de La Frontera. Written informed consent to participate in this study was provided by the participants’ legal guardian/next of kin.

## Author Contributions

JG-N created the research question, conducted bibliographic search, methodological design, contributed to analysis, results, and discussion. KP-L conducted the bibliographic search, theoretical framework, integrated results, and contributed to the discussion. JB-V performed the data collection, contributed to analysis, results, and discussion. All authors contributed to the article and approved the submitted version.

## Conflict of Interest

The authors declare that the research was conducted in the absence of any commercial or financial relationships that could be construed as a potential conflict of interest.
